# Combined phacoemulsification and vitrectomy for proliferative diabetic retinopathy: an increased risk of early recurrence but not long-term neovascular glaucoma

**DOI:** 10.1186/s40942-025-00758-2

**Published:** 2025-11-28

**Authors:** Gengjia Li, Ruibin Wu, Xinyi Zhang, Mingwei Zheng, Qiujie Chen

**Affiliations:** Department of Ophthalmology, The First Affiliated Hospital of Shantou University Medical College, No. 57 Changping Road, Shantou, 515041 China

**Keywords:** Proliferative diabetic retinopathy, Pars plana vitrectomy, Phacoemulsification, Neovascular glaucoma, Vitreous hemorrhage, Surgical outcomes, PSM

## Abstract

**Purpose:**

To compare the long-term incidence of recurrent vitreous hemorrhage(VH) and neovascular glaucoma (NVG) in patients with proliferative diabetic retinopathy (PDR) undergoing pars plana vitrectomy (PPV) alone versus combined PPV and phacoemulsification (PPV&P).

**Methods:**

This large, single-center, retrospective cohort study analyzed 548 eyes (137 PPV&P, 411 PPV-alone) with a minimum 12-month follow-up. Kaplan-Meier survival analysis and multivariable Cox proportional hazards regression models were used to compare outcomes and identify independent predictors for complications within a 2-year follow-up period. A supplementary propensity score-matched (PSM) analysis was also performed to confirm the findings.

**Results:**

Over the entire follow-up, the overall incidence of recurrence and NVG did not differ significantly between the groups. However, multivariable Cox regression analysis for the first two years revealed that PPV-alone was a significant protective factor against recurrence (adjusted Hazard Ratio [aHR], 0.61; 95% CI, 0.40–0.92), while severe preoperative traction was a major risk factor (aHR, 2.24; 95% CI, 1.39–3.60). For NVG development within two years, the surgical approach was not a significant factor. Instead, severe preoperative traction (aHR, 5.88; 95% CI, 1.67–20.68) and older age (aHR, 1.06; 95% CI, 1.01–1.11) were the primary independent risk factors.These findings were confirmed in a 1:1 age-matched PSM cohort.

**Conclusion:**

Combined PPV with phacoemulsification is associated with an increased risk of recurrence within the first two postoperative years but does not increase the risk of neovascular glaucoma. The severity of baseline proliferative disease, particularly the degree of traction, is a more critical determinant of postoperative complications than the surgical strategy regarding the lens. This supports tailoring the surgical approach based on individual patient cataract status and retinal pathology.

**Supplementary Information:**

The online version contains supplementary material available at 10.1186/s40942-025-00758-2.

## Introduction

Diabetic retinopathy (DR) is a leading cause of preventable blindness among working-age adults worldwide [1–4]. Its most advanced stage, proliferative diabetic retinopathy (PDR), is characterized by retinal neovascularization that can lead to vision-threatening complications, including persistent vitreous hemorrhage (VH) and tractional retinal detachment (TRD) [5]. For these severe manifestations, pars plana vitrectomy (PPV) is the definitive surgical intervention, enabling the clearance of hemorrhagic media and the release of vitreoretinal traction [6].

A frequent comorbidity in PDR patients is cataract, creating a clinical dilemma: whether to perform PPV alone or combine it with phacoemulsification (PPV&P) in a single session. The rationale for a combined procedure is strong, offering the convenience of a single surgery, faster visual rehabilitation, and avoidance of the technical challenges inherent in operating on a previously vitrectomized eye [7, 8]. Furthermore, removing the cataract provides the surgeon an unobstructed, panoramic view of the posterior segment, which facilitates more meticulous membrane dissection and complete panretinal endophotocoagulation [7].

However, these benefits are countered by concerns that PPV&P may exacerbate postoperative complications. The extensive surgical manipulation of a combined procedure can intensify the breakdown of the blood-aqueous barrier. This physiological disruption is hypothesized to increase the risk of severe postoperative inflammation, cystoid macular edema, and, most critically, anterior segment neovascularization leading to neovascular glaucoma (NVG)—a devastating and often intractable complication [9–11]. Consequently, the optimal surgical strategy remains a subject of intense debate.

Previous studies on this topic have yielded conflicting and inconclusive results. While some reports suggest combined surgery does not increase the risk of NVG or recurrent VH [12–14], others associate it with poorer outcomes [15, 16]. These discrepancies may stem from variations in study design, patient populations, follow-up duration, and surgical techniques. Critically, a definitive consensus is lacking on whether the surgical approach independently influences the long-term risk of major complications after accounting for baseline patient and disease characteristics.

Therefore, this large-scale, single-center retrospective study was designed to address this critical knowledge gap. By analyzing a substantial cohort of PDR patients with long-term follow-up, we aimed to rigorously compare the incidence of recurrent vitreous hemorrhage and neovascular glaucoma between patients undergoing PPV alone versus combined PPV&P. Using survival analysis and multivariable Cox regression models, we sought not only to compare outcomes but also to identify independent predictors of these vision-threatening complications, thereby providing further evidence to guide clinical decision-making.

## Methods

### Study design and ethical approval

This single-center, retrospective cohort study was conducted at the Department of Ophthalmology, The First Affiliated Hospital of Shantou University Medical College, in adherence with the tenets of the Declaration of Helsinki. The study protocol was approved by the institutional review board, which waived the requirement for informed consent due to the retrospective nature of the analysis.

### Patient selection and study groups

We reviewed the medical records of patients with PDR who underwent either pars PPV alone or PPV&P between January 2019 and May 2024. A total of 548 eyes (137 in the PPV&P group and 411 in the PPV group) met the criteria. Patients were included if they were aged 18 years or older, had surgical indications for PDR (Persistent vitreous hemorrhage or tractional retinal detachment), and had a minimum follow-up of 12 months.

Exclusion criteria were: (1) prior vitrectomy or cataract surgery in the study eye; (2) pre-existing rubeosis iridis or NVG, as determined by slit-lamp examination of the iris and baseline IOP measurement; (3) other significant confounding ocular pathologies, such as age-related macular degeneration (AMD), high myopia (axial length > 26 mm or refractive error > − 6.0 D), or retinal vascular occlusions; (4) incomplete follow-up data; and(5) intraoperative posterior capsule tear or significant zonular complications.

Based on the procedure performed, patients were stratified into two cohorts: the PPV&P group and the PPV group. The PPV&P group included patients with visually significant cataracts. The PPV group consisted of phakic patients with clear or minimally opaque lenses. The decision was at the discretion of the operating surgeon, as detailed in the Limitations.

### Surgical procedures

All surgeries were performed by two experienced vitreoretinal surgeons, each with over 10 years of experience, following a standardized institutional protocol.A standardized 23-gauge three-port PPV was executed on all cases, with scleral incisions sutured postoperatively to ensure wound integrity. The procedure involved a core vitrectomy, induction of posterior vitreous detachment if not already present, and meticulous removal of vitreous and epiretinal membranes using delamination, segmentation, and peeling techniques to relieve all traction. Comprehensive panretinal endophotocoagulation (PRP) was applied from the posterior pole to the ora serrata, with scleral depression as needed to ensure complete peripheral treatment. The choice of intravitreal tamponade—balanced salt solution, air, C3F8 gas, or silicone oil—was at the surgeon’s discretion based on intraoperative findings.

For patients in the PPV&P group, phacoemulsification was performed prior to vitrectomy, followed by the implantation of a foldable acrylic intraocular lens into the capsular bag.Preoperative anti-VEGF injections were administered 3–7 days before surgery at the surgeon’s discretion, typically for cases with active neovascularization.

### Data collection and outcome measures

Relevant preoperative, intraoperative, and postoperative data were extracted from patient medical records. Preoperative variables included demographics, history of diabetes, systemic markers (serum creatinine, HbA1c), ocular history, recent anti-VEGF use, and primary surgical indications. Intraoperative variables included the grades of lens sclerosis and proliferative traction [17], tamponade agent, laser parameters, and any complications. This proliferative traction grading system is adapted from the classification proposed by Kroll et al. [17], incorporating the extent of neovascularization and detachment range based on clinical examination and imaging modalities such as optical coherence tomography (OCT) and fundus photography.

The primary outcomes were the incidence of recurrent vitreous hemorrhage and the development of NVG. Recurrence was defined as any postoperative hemorrhage obscuring the retina for over three weeks. NVG was defined as iris neovascularization with elevated IOP. Secondary outcomes included changes in best-corrected visual acuity (BCVA) and IOP, and rates of postoperative complications like ocular hypertension (IOP > 25 mmHg). Time-to-event was calculated from the surgery date to the diagnosis date of a primary outcome.

### Statistical analysis

Statistical analyses were performed using SPSS software (version 25.0). Continuous variables were compared using independent samples t-tests, while categorical variables were compared using the chi-squared (χ²) or Fisher’s exact test. Time-to-event data for recurrence and NVG were analyzed using the Kaplan-Meier method with the log-rank test for curve comparison. Multivariable Cox proportional hazards regression models were constructed to identify independent predictors for outcomes within a 2-year follow-up period. For all analyses, a two-sided P value of less than 0.05 was considered statistically significant.

To address baseline confounding, particularly age, a supplementary 1:1 PSM analysis was performed. Patients in the PPV&P group were matched to patients in the PPV group based on the logit of the propensity score derived from age. This yielded a matched cohort of 134 pairs (268 eyes). Kaplan-Meier and Cox regression analyses were repeated on this matched cohort to confirm the primary findings. The detailed results of this PSM analysis are provided in the Supplementary Materials.

## Results

### Baseline demographics and clinical characteristics

A total of 548 eyes from 548 patients were included, with 137 in the PPV&P group and 411 in the PPV group. Patients in the PPV&P group were significantly older than those in the PPV group (mean [SD] age, 57.53 [9.59] vs. 50.99 [9.71] years, respectively; *P* < 0.001).

The two groups were well-matched at baseline, with no significant differences in history of diabetes, systemic health markers (serum creatinine, HbA1c), history of prior laser treatment, IOP, BCVA, surgical indications, preoperative anti-VEGF use, or the grade of proliferative traction (*P* > 0.05 for all). As anticipated by the surgical grouping, lens opacity was significantly more severe in the PPV&P group (*P* < 0.001) (Table 1).

**Table 1:** Baseline characteristics and surgery indications

Parameter	PPV&P(*n* = 137)	PPV(*n* = 411)	*P* Value
Age, mean (SD), y	57.53(9.59)	50.99(9.71)	**<0.001**
Sex, No. (%)			
Male	80(58.4)	269(65.5)	0.137
Female	57(41.6)	142(34.5)	
History of diabetes, mean (SD), y	8.69(4.37)	7.95(5.27)	0.143
HbA1c, mean (SD),%	7.40(1.62)	7.48(1.70)	0.640
Creatinine, mean (SD), umol/L	127.99(120.70)	122.31(102.40)	0.592
Preoperative IOP, mean (SD), mmHg	13.16(1.67)	13.21(1.65)	0.781
Preoperative BCVA, Snellen(logMAR), mean (SD)	1.66(0.63)	1.58(0.57)	0.156
Preoperative lens nuclear sclerosis, No. (%)			
Grade 0/1	4(2.9)	312(75.9)	
Grade 2	51(37.2)	95(23.1)	**<0.001**
Grade 3/4	82(59.9)	4(1.0)	
Preoperative Grading of Traction, No. (%)			
Severe	34(24.8)	111(27.0)	
Mild or moderate	67(48.9)	190(46.2)	0.939
No traction	36(26.3)	110(26.8)	
Previous laser, No. (%)	58(42.3)	140(34.1)	0.081
Previous anti‑VEGF within 2 weeks, No. (%)	104(78.1)	339(82.5)	0.091
Surgery indications, No. (%)			
VH	90(65.7)	262(63.7)	0.681
TRD ± VH	47(34.3)	149(36.3)	

Nuclear sclerosis was assessed at the slit lamp and classified as follows: Grade 0: Clear lens.Grade 1: Early nuclear sclerosis with mild yellow discoloration of the posterior lens in the slit beam.Grade 2: Yellow discoloration throughout the lens.Grade 3: Yellow-brown discoloration throughout the lens.Grade 4: Brown discoloration of the entire lens.Preoperative proliferative traction was graded as no traction(complete posterior vitreous detachment), mild (localized fibrosis without macular involvement), moderate (multifocal fibrosis with partial macular traction), or severe (extensive fibrosis with macular detachment or broad RD), based on clinical and imaging assessment, incorporating extent of neovascularization and detachment range

Abbreviation: y,years; PPV&P,Pars plana vitrectomy with cataract phacoemulsification; HbA1c, Glycosylated hemoglobin; IOP, Intraocular Pressure; LogMAR, Logarithm of the Minimum Angle of Resolution; BCVA, Best Corrected Visual Acuity; VH, vitreous hemorrhage; TRD, tractional retinal detachment; VEGF, vascular endothelial growth factor

### Surgical and postoperative outcomes

Intraoperative management, including the choice of tamponade agent, application of retinal laser, and use of intravitreal triamcinolone, was comparable between groups (*P* > 0.05). However, the PPV&P group experienced a significantly higher rate of intraoperative complications, including anterior chamber hemorrhage and corneal edema (*P* < 0.01 for all) (Table 2).

**Table 2:** Surgery procedures and intraoperative complications

Parameter	PPV&P(*n* = 137)	PPV(*n* = 411)	*P* Value
Surgery Procedures			
Endotamponade, No. (%)			
BSS	78(56.9)	244(59.4)	
Air	6(4.4)	13(3.2)	0.857
Silicone Oil	38(27.7)	115(28.0)	
C3F8 gas	15(10.9)	39(9.5)	
Endolaser, No. (%)	132(96.4)	406(98.8)	0.065
TA injection, No. (%)	43(31.4)	100(24.3)	0.103
Intraoperative Complications, No. (%)			
Anterior chamber hemorrhage	6(4.4)	1(0.2)	**0.001**
Corneal edema	9(6.6)	1(0.2)	**<0.001**

Abbreviation: PPV&P,Pars plana vitrectomy with cataract phacoemulsification; BSS, balanced salt solution; TA, triamcinolone acetonide

The mean follow-up duration was 29.63 [12.04] months for the PPV&P group and 30.49 [11.80] months for the PPV group. Postoperative outcomes were largely similar, with no significant differences in final IOP, BCVA improvement, or the overall rates of recurrence of VH (29.9% vs. 27.0%), NVG (8.0% vs. 7.8%), or the need for repeat PPV (17.5% vs. 13.1%) (*P* > 0.05 for all). However, the PPV group had a significantly better BCVA (1.01 logMAR vs. 1.16 logMAR in the PPV&P group, *P* = 0.027) (Table 3).

**Table 3:** Postoperative IOP, BCVA and complications

Parameter	PPV&P(*n* = 137)	PPV(*n* = 411)	*P* Value
Follow-up time, mean (SD), y	29.63(12.04)	30.49(11.80)	0.461
Postoperative IOP, mean (SD), mmHg	14.91(5.39)	14.60(5.16)	0.542
Postoperative BCVA, Snellen(logMAR), mean (SD)	1.16(0.76)	1.01(0.63)	**0.027**
BCVA Improvement (ETDRS letters)	25.10(36.81)	28.33(36.86)	0.375
Complications			
Recurrence (VH), No. (%)#	41(29.9)	111(27.0)	0.509
Time after surgery, mean (SD), m	10.04(11.53)	13.81(10.57)	0.058
HbA1c, mean (SD),%	7.10(1.50)	7.58(1.50)	0.079
Creatinine, mean (SD), umol/L	137.34(114.43)	130.83(113.17)	0.754
NVG, No. (%)	11(8.0)	32(7.8)	0.927
Time after surgery, mean (SD), m	27.96(12.58)	23.00(10.57)	0.208
HbA1c, mean (SD),%	7.61(1.39)	7.73(1.75)	0.844
Creatinine, mean (SD), umol/L	120.82(70.26)	98.69(41.58)	0.214
Others, No. (%)			
Corneal edema	15(10.9)	30(7.3)	0.178
Silicone oil in chamber	6(4.4)	3(0.7)	**0.004**
Inflammatory reaction	8(5.8)	7(1.7)	**0.010**
Ocular hypertension	21(15.3)	80(19.5)	0.280
Macular edema	19(13.9)	46(11.2)	0.401
Recurrence of RD	17(12.4)	38(9.2)	0.286
Repeat PPV	24(17.5)	54(13.1)	0.204

#, Defined as any postoperative hemorrhage sufficient to obscure the retina and cause vision loss lasting more than three weeks

Abbreviation: PPV&P,Pars plana vitrectomy with cataract phacoemulsification; IOP, Intraocular Pressure; LogMAR, Logarithm of the Minimum Angle of Resolution; BCVA, Best Corrected Visual Acuity; y,years; m,months; ETDRS, Early Treatment Diabetic Retinopathy Study Chart; VH, vitreous hemorrhage; RD, Retinal detachment; HbA1c, Glycosylated hemoglobin; NVG, Neovascular glaucoma

Additionally, the PPV&P group had a significantly higher incidence of postoperative inflammatory reactions (5.8% vs. 1.7%) and silicone oil in the anterior chamber (4.4% vs. 0.7%) (Table 3).

### Time-to-event analysis

**Fig. 1:**
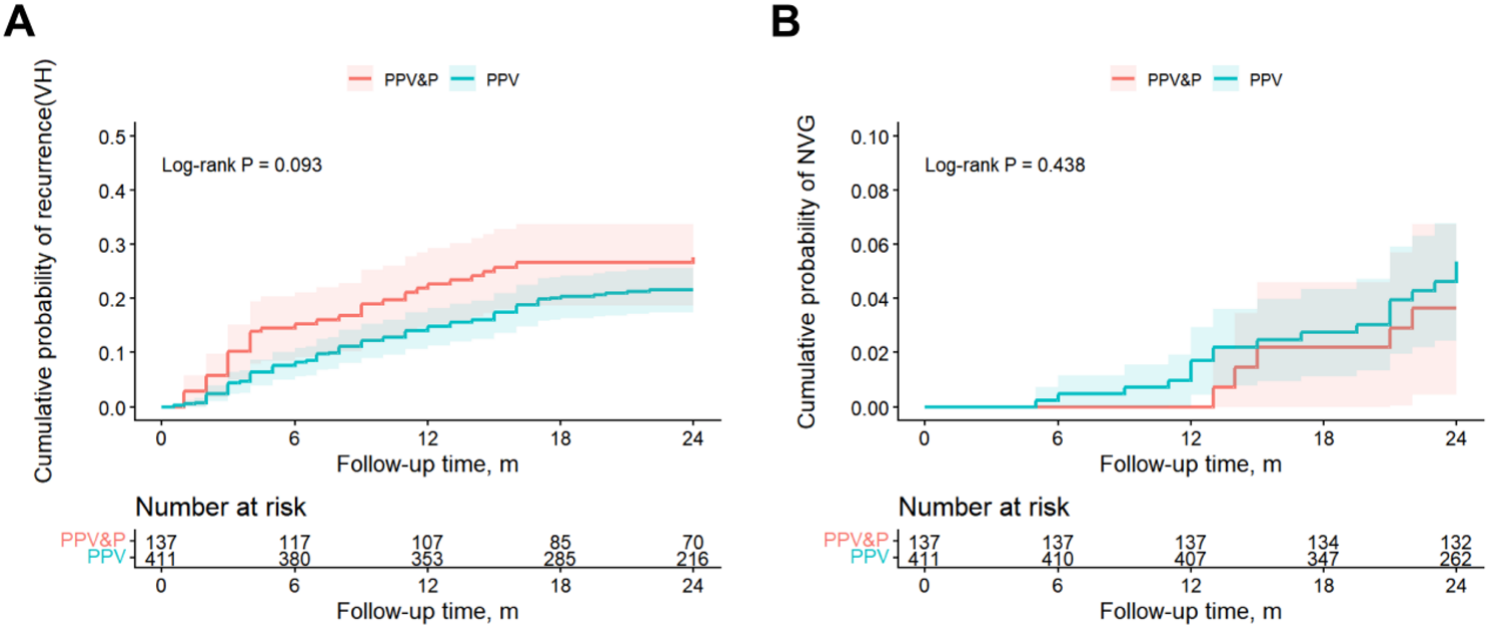
Kaplan-Meier survival analysis of postoperative complications within two years. Comparison of the cumulative incidence of (**A**) recurrent vitreous hemorrhage(VH) and (**B**) neovascular glaucoma (NVG) between the combined phacoemulsification-vitrectomy (PPV&P) and vitrectomy-alone (PPV) groups. Shaded areas represent 95% confidence intervals (CIs). The number of patients at risk at various time points is detailed below the x-axis

Kaplan-Meier analysis revealed no significant difference in the overall cumulative risk of recurrence (log-rank *P* = 0.093) or NVG (log-rank *P* = 0.438) between the groups over the 2-year follow-up period (Fig. 1). The estimated 2-year risk for recurrence was 27.6% in the PPV&P group and 21.6% in the PPV group. The 2-year risk for NVG was 3.7% in the PPV&P group and 5.4% in the PPV group.

### Independent predictors of postoperative complications

Multivariable Cox regression analysis was performed for the 2-year follow-up period to identify independent predictors of major complications.

**Fig. 2:**
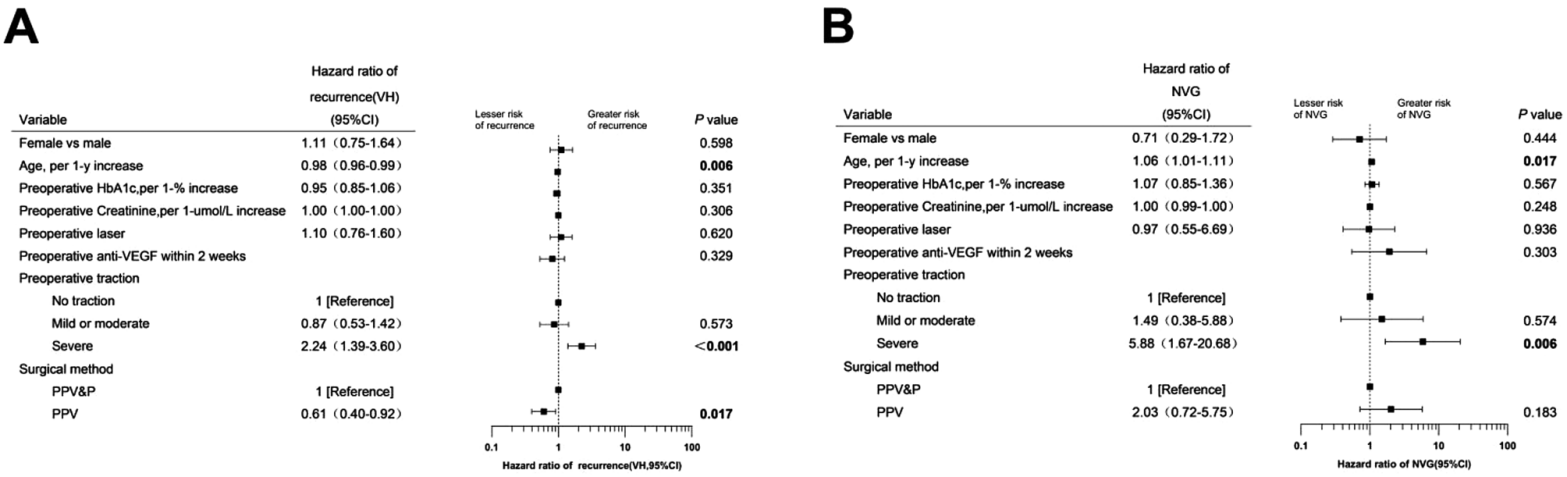
Forest plots of multivariable cox regression analysis for complications within two years. The plots illustrate the adjusted hazard ratios (aHRs) for predictors of (**A**) recurrence (VH) and (**B**) neovascular glaucoma (NVG) within a 2-year postoperative period. Data points represent the aHRs, and horizontal lines indicate the 95% confidence intervals (CIs). The vertical dashed line at an HR of 1.0 signifies no effect. Predictors with CIs that do not cross this line are considered statistically significant

For recurrence, significant predictors included undergoing PPV surgery (adjusted Hazard Ratio [aHR], 0.61; 95% CI, 0.40–0.92), which was protective, and older age (aHR, 0.98; 95% CI, 0.96–0.99), which was also protective. Conversely, the presence of severe preoperative traction was a significant risk factor (aHR, 2.24; 95% CI, 1.39–3.60) (Fig. 2A). For the development of NVG, the surgical approach was not a significant factor. The primary risk factors were the presence of severe preoperative traction (aHR, 5.88; 95% CI, 1.67–20.68) and older age (aHR, 1.06; 95% CI, 1.01–1.11) (Fig. 2B).

### Confirmatory propensity score-matched analysis

A 1:1 age-matched PSM analysis yielded 134 pairs with well-balanced baseline characteristics (Supplementary Table 1). Lens opacity was significantly more severe in the PPV&P group as well (*P* < 0.001). Intraoperative management was comparable between groups (*P* > 0.05). The PPV&P group experienced a significantly higher rate of corneal edema (Supplementary Table 2). Postoperative IOP, BCVA, and complications after PSM showed no significant differences between groups, except for the recurrence time, which was longer in the PPV group (10.04 [11.53] months vs. 15.67 [11.54] months, *P* < 0.05) (Supplementary Table 3).

Kaplan-Meier analysis revealed no significant difference in the overall cumulative risk of recurrence (log-rank *P* = 0.063) or NVG (log-rank *P* = 0.407) between the groups over the 2-year follow-up period (Supplementary Fig. 1). The estimated 2-year risk for recurrence was 28.2% in the PPV&P group and 19.3% in the PPV group. The 2-year risk for NVG was 3.7% in the PPV&P group and 6.0% in the PPV group.

Analysis of this matched cohort confirmed our primary findings. Undergoing PPV surgery was a significant predictor for recurrence (adjusted hazard ratio [aHR], 0.58; 95% CI, 0.35–0.99). However, no significant difference was observed in the risk of NVG (aHR, 2.03; 95% CI, 0.64–6.48) (Supplementary Fig. 2).

## Discussion

This study contributes nuanced evidence to the long-standing debate over the optimal surgical management for PDR with coexisting cataracts. Our primary finding is that while the overall, long-term incidence of major complications did not differ between combined phacoemulsification with pars plana vitrectomy (PPV&P) and PPV, a time-dependent analysis revealed a critical distinction: combined surgery was associated with a significantly higher risk of recurrence specifically within the first two postoperative years.

### Recurrent vitreous hemorrhage: an early risk for combined surgery

Our multivariable Cox regression analysis identified PPV-alone as an independent protective factor against recurrence within a 2-year timeframe (aHR, 0.61). This result substantiates the hypothesis that the greater surgical trauma of a combined procedure potentiates a more significant breakdown of the blood-ocular barrier, leading to a transient, heightened inflammatory state. This early postoperative period is critical, as fragile neovascular tissue is most susceptible to bleeding [5]. The significantly higher incidence of postoperative inflammatory reactions observed in our PPV&P group lends clinical support to this pathophysiological mechanism.

Interestingly, our Kaplan-Meier analysis showed this increased risk was front-loaded. Beyond three years, the recurrence curve for the PPV&P group flattened, while the risk in the PPV group continued to ascend gradually. Although long-term data must be interpreted cautiously due to patient attrition, this trend suggests that once the initial inflammation from combined surgery resolves, the re-bleeding risk diminishes. Conversely, patients who undergo PPV may face a delayed risk from subsequent cataract progression, which can impede fundus visualization for monitoring and supplementary laser, potentially allowing undetected neovascular activity to cause late-onset hemorrhage.

An important finding is that this increased early risk was specific to recurrent VH and did not translate to an increased risk of NVG. We hypothesize this is due to distinct pathophysiological mechanisms. Early recurrent VH is often a result of transient inflammation and blood-ocular barrier breakdown, which is exacerbated by the increased trauma of combined surgery. In contrast, NVG is driven by a sustained, high ischemic load. Our findings suggest that complete vitrectomy and thorough endophotocoagulation effectively suppress this ischemic drive long-term, mitigating the risk of NVG regardless of lens status.

### Neovascular glaucoma: challenging traditional dogma

A pivotal finding of our study is the lack of any significant association between combined surgery and an increased risk of NVG. For decades, a primary argument against PPV&P has been the fear that removing the crystalline lens [12]—a supposed barrier to the anterior diffusion of vasoproliferative factors like VEGF—would precipitate catastrophic anterior segment neovascularization [14, 18–22]. Our results, consistent with several contemporary studies [12–14], challenge this dogma. The 2-year NVG risks were low and statistically indistinguishable between groups. This suggests that with modern surgical techniques—including thorough removal of the vitreous scaffold, complete relief of traction, and extensive panretinal endophotocoagulation—the theoretical risk conferred by phacoemulsification is effectively negated.

Instead of surgical strategy, our analysis identified severe preoperative traction and older age as the most potent independent predictors for NVG. This underscores a crucial paradigm shift: the primary driver of NVG is not the absence of the lens but the severity of the underlying retinal ischemia. Incomplete traction removal or insufficient photocoagulation leaves a powerful ischemic stimulus intact, which is the true engine of neovascularization. The association with older age is a novel finding that may reflect a reduced vasoregenerative capacity or increased endothelial susceptibility to ischemic insults in elderly patients.

### Clinical implications and surgical decision-making

Our findings have direct implications for clinical practice, supporting a move away from a rigid, risk-averse stance on PPV&P toward a more individualized approach [6, 7, 23].

For patients with visually significant cataracts, the benefits of a combined procedure—superior intraoperative visualization facilitating more complete surgery and faster visual rehabilitation—likely outweigh the transiently increased risk of early postoperative recurrence, especially since our data show the long-term risk of NVG is not elevated.

For patients with a clear or only mildly opaque lens, performing PPV-alone may be the more prudent approach to minimize the risk of early recurrent hemorrhage, with a staged phacoemulsification performed later if needed.

Regardless of the approach, the surgical focus must remain on addressing the fundamental pathology. Meticulous removal of all vitreoretinal traction [7, 13, 24, 25] and comprehensive panretinal photocoagulation are paramount in preventing both recurrent VH and NVG [26–29]. The severity of preoperative traction, as identified in our study, should be a primary factor in guiding surgical strategy and patient counseling.

It is important to note that severe preoperative traction is often a clinical surrogate for advanced, long-standing retinal ischemia. While ischemia is the fundamental biological driver of neovascularization, severe traction likely contributes as both a marker of this ischemic severity and an anatomical barrier to complete panretinal photocoagulation.

### Limitation

This study has several limitations. First, its retrospective, single-center design is susceptible to inherent selection and ascertainment bias. Although we performed a PSM analysis to account for age, other unmeasured confounders remain. Second, the surgical decision was not randomized, leading to inherent differences between groups.Third, all surgeries were performed by two experienced surgeons. While this standardizes technique, it also introduces potential inter-surgeon variability, and our results may not be generalizable to less experienced surgeons. Fourth, we did not perform a subgroup analysis on treatment-naïve patients, and the unsystematic use of preoperative, intraoperative, or postoperative anti-VEGF therapy is a potential confounder.Fifth, we lacked quantitative data on the preoperative retinal ischemic load, such as from widefield fluorescein angiography, which is a primary driver of complications. Sixth, we did not systematically collect data on the need for supplementary postoperative PRP. Seventh, while the follow-up was substantial, it may be insufficient to capture very late-onset complications. Finally, anterior segment assessments did not routinely include advanced imaging like ultrasound biomicroscopy (UBM), which could have provided more detailed evaluation of potential associations between anterior hyaloid proliferation (AHP), iris neovascularization (NVI), and intraocular pressure (IOP) changes. Therefore, a large-scale, multicenter randomized controlled trial is still necessary to definitively confirm these findings and provide the highest level of evidence.

## Conclusion

In conclusion, this study demonstrates that while combined PPV and phacoemulsification is associated with an increased risk of recurrent vitreous hemorrhage within the first two postoperative years, it does not increase the long-term risk of neovascular glaucoma. The most critical determinant of postoperative outcomes is not the surgical strategy for the lens, but rather the severity of the baseline proliferative disease and the thoroughness of the vitrectomy and endophotocoagulation. This evidence should empower surgeons to tailor their approach based on the individual patient’s cataract status and retinal pathology, rather than an unsubstantiated fear of devastating complications from a combined procedure.

## Supplementary Information

Below is the link to the electronic supplementary material.

Supplementary Material 1

Supplementary Material 2

Supplementary Material 3

Supplementary Material 4

Supplementary Material 5

## Data Availability

The data supporting the findings of this study are available from the corresponding author upon reasonable request. Due to their size, the raw datasets cannot be deposited in a public repository but can be accessed following a justified inquiry.
