# Supplementary material for: Combined phacoemulsification and vitrectomy for proliferative diabetic retinopathy: an increased risk of early recurrence but not long-term neovascular glaucoma
Source: Int J Retina Vitreous. 2025 Nov 28;11:130. doi: 10.1186/s40942-025-00758-2 (PMC12661772; doi:10.1186/s40942-025-00758-2)
Supplement: Supplementary file 1 — Supplementary Material 1 [file 40942_2025_758_MOESM1_ESM.docx]

Supplymentary Table 1.Baseline Characteristics and Surgery Indications after PSM

| Parameter | PPV&P（n=134） | PPV（n=134） | *P* Value |
| --- | --- | --- | --- |
| **Age, mean (SD), y** | 57.15（9.34） | 55.58（10.34） | 0.194 |
| **Sex, No. (%)** |  |  |  |
| Male | 79（59.0） | 88（65.7） | 0.257 |
| Female | 55（41.0） | 46（34.3） |  |
| History of diabetes**, mean (SD), y** | 8.75（4.34） | 9.16（6.61） | 0.556 |
| HbA1c, mean (SD),% | 7.37（1.62） | 7.57（1.88） | 0.336 |
| **Creatinine**, mean (SD), umol/L | 128.29（121.86） | 118.85（112.81） | 0.511 |
| **Preoperative IOP, mean (SD),** mmHg | 13.15（1.68） | 13.23（1.74） | 0.668 |
| **Preoperative** BCVA,Snellen(logMAR)**, mean (SD)** | 1.65（0.63） | 1.58（0.56） | 0.313 |
| Preoperative lens nuclear sclerosis**, No. (%)** |  |  |  |
| Grade 0/1 | 4（2.9） | 90（67.2） |  |
| Grade 2 | 51（38.1） | 42（31.3） | **＜0.001** |
| Grade 3/4 | 79（59.0） | 2（1.5） |  |
| Preoperative Grading of Traction**, No. (%)** |  |  |  |
| Severe | 33（24.6） | 38（28.4） |  |
| Mild or moderate | 67（50.0） | 54（40.3） | 0.274 |
| No traction | 34（25.4） | 42（31.3） |  |
| Previous laser**, No. (%)** | 58（43.2） | 45（33.6） | 0.103 |
| Previous anti‑VEGF within 2 weeks**, No. (%)** | 101（75.3） | 110（82.1） | 0.179 |
| Surgery indications**, No. (%)** |  |  |  |
| VH | 88（65.7） | 88（65.7） | 1.000 |
| TRD±VH | 46（34.3） | 46（34.3） |  |

Nuclear sclerosis was assessed at the slit lamp and classified as follows:Grade 0: Clear lens.Grade 1: Early nuclear sclerosis with mild yellow discoloration of the posterior lens in the slit beam.Grade 2: Yellow discoloration throughout the lens.Grade 3: Yellow-brown discoloration throughout the lens.Grade 4: Brown discoloration of the entire lens.Preoperative proliferative traction was graded as no traction(complete posterior vitreous detachment), mild (localized fibrosis without macular involvement), moderate (multifocal fibrosis with partial macular traction), or severe (extensive fibrosis with macular detachment or broad RD), based on clinical and imaging assessment, incorporating extent of neovascularization and detachment range.

Abbreviation：PSM,Propensity Score Matching;y,years;PPV&P,Pars plana vitrectomy with cataract phacoemulsification; HbA1c,Glycosylated hemoglobin;IOP,Intraocular Pressure;LogMAR,Logarithm of the Minimum Angle of Resolution;BCVA,Best Corrected Visual Acuity;VH,vitreous hemorrhage; TRD, tractional retinal detachment; VEGF, vascular endothelial growth factor.
