# Supplementary material for: Combined phacoemulsification and vitrectomy for proliferative diabetic retinopathy: an increased risk of early recurrence but not long-term neovascular glaucoma
Source: Int J Retina Vitreous. 2025 Nov 28;11:130. doi: 10.1186/s40942-025-00758-2 (PMC12661772; doi:10.1186/s40942-025-00758-2)
Supplement: Supplementary file 2 — Supplementary Material 2 [file 40942_2025_758_MOESM2_ESM.docx]

Supplymentary Table 2.Surgery Procedures and Intraoperative Complications after PSM

| Parameter | PPV&P（n=134） | PPV（n=134） | *P* Value |
| --- | --- | --- | --- |
| Surgery Procedures |  |  |  |
| Endotamponade**, No. (%)** |  |  |  |
| BSS | 76（56.7） | 81（60.4） |  |
| Air | 6（4.4） | 6（4.4） | 0.763 |
| Silicone Oil | 37（27.6） | 37（27.6） |  |
| C3F8 gas | 15（11.1） | 10（7.4） |  |
| Endolaser**, No. (%)** | 129（96.3） | 132（98.5） | 0.447 |
| TA injection**, No. (%)** | 43（32.1） | 41（30.6） | 0.792 |
| Intraoperative Complications**, No. (%)** |  |  |  |
| Anterior chamber hemorrhage | 5（3.7） | 1（0.7） | 0.213 |
| Corneal edema | 9（6.7） | 1（0.7） | **0.019** |

Abbreviation:PSM,Propensity Score Matching;PPV&P,Pars plana vitrectomy with cataract phacoemulsification; BSS,balanced salt solution;TA, triamcinolone acetonide.
