# Supplementary material for: Combined phacoemulsification and vitrectomy for proliferative diabetic retinopathy: an increased risk of early recurrence but not long-term neovascular glaucoma
Source: Int J Retina Vitreous. 2025 Nov 28;11:130. doi: 10.1186/s40942-025-00758-2 (PMC12661772; doi:10.1186/s40942-025-00758-2)
Supplement: Supplementary file 3 — Supplementary Material 3 [file 40942_2025_758_MOESM3_ESM.docx]

Supplymentary Table 3.Postoperative IOP, BCVA and Complications after PSM

| Parameter | PPV&P（n=134） | PPV（n=134） | *P* Value |
| --- | --- | --- | --- |
| Follow-up time**, mean (SD), y** | 29.78（12.05） | 29.96（11.74） | 0.900 |
| **Postoperative IOP, mean (SD),** mmHg | 14.93（5.45） | 15.06（5.95） | 0.860 |
| **Postoperative** BCVA,Snellen(logMAR)**, mean (SD)** | 1.15（0.76） | 1.06（0.62） | 0.258 |
| BCVA Improvement (ETDRS letters) | 26.01（37.20） | 26.13（38.05） | 0.808 |
| Complications |  |  |  |
| Recurrence (VH)**, No. (%)#** | 41（30.6） | 36（26.9） | 0.500 |
| Time after surgery**, mean (SD), m** | 10.04（11.53） | 15.67（11.54） | **0.036** |
| HbA1c, mean (SD),% | 7.10（1.50） | 7.44（1.43） | 0.321 |
| **Creatinine**, mean (SD), umol/L | 137.34（114.43） | 143.81（152.93） | 0.833 |
| NVG**, No. (%)** | 11（8.2） | 13（9.7） | 0.668 |
| Time after surgery**, mean (SD), m** | 27.96（12.58） | 24.12（10.44） | 0.422 |
| HbA1c, mean (SD),% | 7.61（1.39） | 7.99（2.23） | 0.631 |
| **Creatinine**, mean (SD), umol/L | 120.82（70.26） | 103.69（46.10） | 0.481 |
| **Others, No. (%)** |  |  |  |
| Corneal edema | 15（11.2） | 9（6.7） | 0.199 |
| **Silicone oil in chamber** | 6（4.5） | 2（1.5） | 0.282 |
| Inflammatory reaction | 8（6.0） | 3（2.2） | 0.124 |
| Ocular hypertension | 21（15.7） | 26（19.4） | 0.422 |
| Macular edema | 19（14.2） | 15（11.2） | 0.463 |
| Recurrence of RD | 17（12.7） | 10（7.5） | 0.155 |
| Repeat PPV | 24（17.9） | 16（11.9） | 0.170 |

#, Defined as any postoperative hemorrhage sufficient to obscure the retina and cause vision loss lasting more than three weeks.

Abbreviation:PSM,Propensity Score Matching;PPV&P,Pars plana vitrectomy with cataract phacoemulsification; IOP,Intraocular Pressure;LogMAR,Logarithm of the Minimum Angle of Resolution;BCVA,Best Corrected Visual Acuity;y,years;m,months;ETDRS,Early Treatment Diabetic Retinopathy Study Chart;VH,vitreous hemorrhage;RD,Retinal detachment;HbA1c,Glycosylated hemoglobin;NVG,Neovascular glaucoma.
