# Supplementary figures and images for: Combined phacoemulsification and vitrectomy for proliferative diabetic retinopathy: an increased risk of early recurrence but not long-term neovascular glaucoma
Source: Int J Retina Vitreous. 2025 Nov 28;11:130. doi: 10.1186/s40942-025-00758-2 (PMC12661772; doi:10.1186/s40942-025-00758-2)

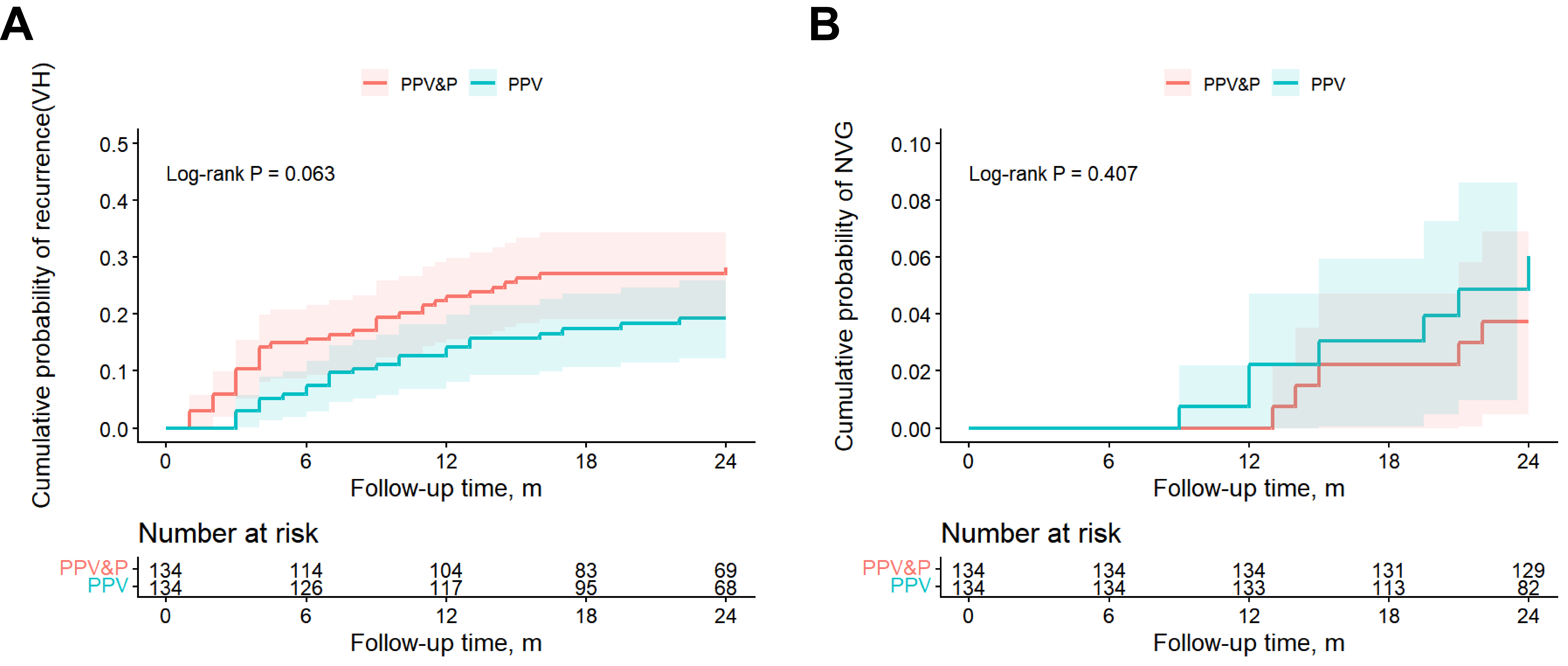

Supplement: Supplementary file 4 — Supplementary Material 4 [file 40942_2025_758_MOESM4_ESM.tif]

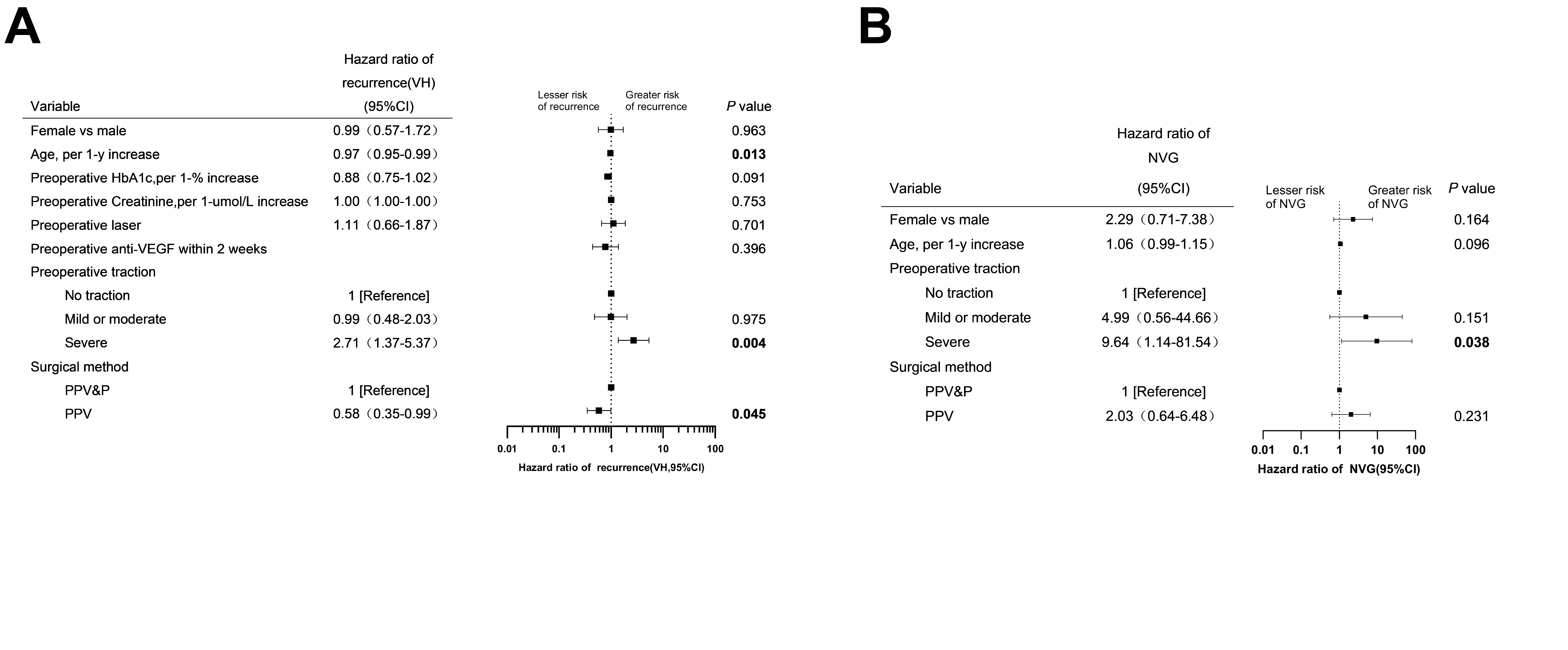

Supplement: Supplementary file 5 — Supplementary Material 5 [file 40942_2025_758_MOESM5_ESM.tif]
